# Variation in quality of grains used in malting and brewing

**DOI:** 10.3389/fpls.2023.1172028

**Published:** 2023-06-12

**Authors:** Glen P. Fox, Harmonie M. Bettenhausen

**Affiliations:** ^1^ Department of Food Science and Technology, University of California, Davis, Davis, CA, United States; ^2^ Center for Craft Food and Beverage, Hartwick College Center for Craft Food and Beverage, Oneonta, NY, United States

**Keywords:** cereal, malting, brewing, metabolomics, proteomics, quality

## Abstract

Cereal grains have been domesticated largely from food grains to feed and malting grains. Barley (*Hordeum vulgare L.*) remains unparalleled in its success as a primary brewing grain. However, there is renewed interest in “alternative” grains for brewing (and distilling) due to attention being placed on flavor, quality, and health (i.e., gluten issues) aspects that they may offer. This review covers basic and general information on “alternative grains” for malting and brewing, as well as an in-depth look at several major biochemical aspects of these grains including starch, protein, polyphenols, and lipids. These traits are described in terms of their effects on processing and flavor, as well as the prospects for improvement through breeding. These aspects have been studied extensively in barley, but little is known about the functional properties in other crops for malting and brewing. In addition, the complex nature of malting and brewing produces a large number of brewing targets but requires extensive processing, laboratory analysis, and accompanying sensory analysis. However, if a better understanding of the potential of alternative crops that can be used in malting and brewing is needed, then significantly more research is required.

## Background

Cereal grains have been one of the major raw materials used in brewing beer for over 10,000 years. Depending upon the region of the world, when humans started to farm with their native grain species, there have been evolutionary changes in the physical size, shapes, and appearance of grains. Regardless of the grain species, the inherent composition is very similar. There are two main parts of the grain, (i) the embryo that grows into a new plant and (ii) the starch endosperm that serves as the storage compartment for starch and proteins. The endosperm has hundreds of small cells that contain starch and proteins, some phenolic acids and lipids. All of these provide food to the growing embryo when germination starts.

## Use of malted barley grain in the beer industry: the brewing process

The brewing process and the creation of beer is a complex process with many important steps and several raw ingredients (water, hops, malt, yeast, etc.) that are either processed or remain alive. As this has been a widely studied and written-about topic, this section aims to be brief and focused on the subject of barley and malt. Every step in this process, like the malting process, is closely regulated in order to create the “big picture” product at the end. However, there are also many variations on the process that lead to alterations in raw ingredients, how they interact, and how we perceive them.

After barley grain is malted (discussed below), the finished malt is milled (similarly to flour) so that it increases the surface area, allowing it to dissolve well in water and allowing more access for enzymatic activity during the mashing step. This process serves to separate the husk from the endosperm and to expose interior starch. Once milled, it is known as “grist.” The grist (which still contains endogenous amylases) is mixed with water (this mixture is known as “mash”) and heated for several hours to encourage a process known as “saccharification” during which the remaining barley starches are converted to simple sugars. The step during which saccharification occurs is known as “mashing,” and immediately following it, the liquids are separated from the solids. Solids are discarded or repurposed, whereas the liquid, known as “wort,” will undergo further processing to become beer. Wort is loosely defined as “an aqueous medium comprising mainly fermentable sugars derived usually from starch-rich cereals but also assimilable nitrogen, oxygen, sources of sulfur and phosphates, the vitamin biotin, calcium, and magnesium ions, together with trace elements such as copper and zinc” (Tester and Morrison, 1992; Mua and Jackson, 1995). The proportions of these components will depend on the composition of the raw ingredients. Malt is rich in these necessary components for yeast health and growth. Normally, wort contains 70%–75% fermentable sugars, composed of glucose, fructose, sucrose, maltose, and maltotriose. The remaining carbohydrates are considered “unfermentable,” meaning they are longer-chain saccharides and branched polymers, unable to be broken down and made available for yeast (Tester and Morrison, 1992; Mua and Jackson, 1995). Free amino nitrogen (FAN), discussed in detail below, is influenced by the variation in raw materials, specifically malt, which is the primary source of FAN for yeast. Understanding malt chemistry and composition is imperative to understanding and manipulating how carbohydrates and proteins are utilized in the process.

The wort is collected from the spent grains and continues to the next step: boiling. Boiling the wort is an essential part of the process, and the way it is performed affects the final beer quality and flavor. Of principal importance in the boil is isomerization and subsequent solubilization of the bitter substance alpha acid from added hops. Boiling the wort also pasteurizes it, freeing it from bacterial contamination. Enzyme activity is halted, and therefore, the remaining saccharide composition of the wort and hence the dextrin content of the final beer is set. Proteins which become insoluble through destruction of structure form a solid precipitate called the “trub,” which is removed from the wort prior to fermentation. The wort is then clarified and chilled. After cooling, brewers add yeast which sill ferment the solubilized simple sugars into alcohol and carbon dioxide.

## Utility of malted barley grain in the beer industry: macrolevel malt chemistry

To deem barley acceptable for the malting process, certain quality parameters are assessed; quality parameters for malting barley are distinct from food barley. Malting is a process by which cereal grains are germinated and partially broken down (“modification”) in such a way that their starch becomes available for conversion to simple sugars. These simple sugars are the substrate for fermentation of beer and distilled spirits; although many grains can be used, barley is the most common raw material for the brewing and distilling industry due to chemical properties that make it a highly efficient and uniform malting grain. The primary function of a barley seed is to produce a mature barley plant. Malting takes advantage of the chemical signals associated with this function to release simple sugars, as described above. The first step in malting is to assess and utilize only prime barley that not only has excelled in the field but can create quality malt with novel or consistent attributes, depending on the end use. There are a few grain quality parameters the barley should fulfill regardless of malting style, such as moisture. Moisture in raw barley grain should not exceed 12% (Chi et al., 2003). This prevents the growth of mold and decreases the likelihood of mycotoxin buildup within the stored grain. Uniform germination is key so that grain modification, which refers to enzymatic degradation of cell walls (by enzymes released from the aleurone layer/scutellum) to convert starches to sugars in the endosperm, is consistent throughout the batch. Nitrogen and protein contents are important attributes in assessing grain quality (Zhang et al., 2017). Acceptable nitrogen and protein levels in two-row malting barley (most common in North America) are less than 2% nitrogen and 11.0%–13.0% protein. Low nitrogen content does not fulfill the growth and maturation needs of yeast during fermentation, whereas high protein content decreases the volume of starch within the endosperm cavity, therefore decreasing the potential extract (Kossmann and Lloyd, 2000). The protein content of the barley grain affects the metabolomic composition and enzyme levels of malt, which is important for the brewer to be aware of, as high protein limits starch degradation, affects mouthfeel and foam stability, and decreases extract available to the brewer. Low protein limits enzymatic activity, and modification is difficult. Hot and dry environments (such as in Colorado) tend to result in higher protein (Yu et al., 2017). The barley must not show signs of disease, preharvest sprout damage, and damage from insects or from fertilizers or pesticides. It is only when the raw barley is deemed acceptable upon inspection and analysis that the stages of malting can begin. Barley must also have a low prevalence of the mycotoxin deoxynivalenol (DON), which is the result of fusarium head blight (FHB) in barley. Fusarium head blight (FHB), characterized by premature bleaching on the “heads” of grains, is caused by the Fusarium fungus that grows on cereal grains such as wheat, barley, oats, and corn. This fungus is encouraged by wet and humid weather and is therefore more common in temperate climates. It produces the mycotoxin deoxynivalenol (DON), or vomitoxin, which causes nausea and vomiting if consumed at high enough levels in humans and livestock and is problematic to the maltster and brewer, leading to potential problems in malting and brewing. Both barley and malt from the same lot should undergo testing because barley DON levels are not correlated with malt DON levels. The FDA has set “advisory limits” of DON on finished wheat products to be 1 ppm, but no limits are set for malt or beer.

## Malting: how macrolevel chemistry is achieved from barley grain

The phenotype of the raw barley (i.e., protein, moisture content) determines the process and outcome of malting, and it has a major impact on flavor and flavor stability during brewing. The metabolites in malted barley, such as nitrogenous compounds (amines), carbohydrates, amino acids, and lipids (fatty acids and their esters), and the interactions they have with hops, water, and yeast under the conditions of brewing influence the flavor in beer and subsequent stability. Steeping, the first step in malting barley, involves partially immersing dried barley grain in water (at 14°C–16°C) to increase moisture content (to a final moisture content of 42%–48%), which stimulates germination. This stage takes 24–48 h and requires turning and venting of the steeping grains (for oxygen replenishment and carbon dioxide release) (Zhang et al., 2017; Bettenhausen et al., 2018). Depending on the maltster, time of year, and barley genotype, the specifics and timing of these steps often vary. Once the “chit” (coleorhiza) has emerged from the grain, the barley is moved to germination beds. These beds maintain a specific temperature (between 16°C and 20°C) and aeration level (usually by auger) with the goal of stimulating enzymes that initiate or protein degradation and hydrolyzation of starch, also known as endosperm “modification”. There are several enzymes released into action during germination. One suite of enzymes degrades β-glucan (β-glucanase), for example. Excess β-glucan increases viscosity in wort (hot water mixed with ground malt) and is an undesirable characteristic in beer. During the starch modification that occurs during germination, α- and β-amylase are both released. These are essential enzymes which break up the native barley starches amylopectin and amylose. This starch breakdown results in glucose chains of varying lengths, including glucose, maltose, maltotriose, and other saccharides, for downstream consumption by yeast during fermentation. Kilning is the last stage in malting and is highly variable. Over the course of 24–30 h, the now “green” malt is dried and cured to affect a roasted flavor and reduce grain moisture level. Kilning temperature is incrementally increased over the course of several hours to achieve the style of malt desired (Zhang et al., 2017; Bettenhausen et al., 2018).

Over- or under-modification can result in beer with too much or too little protein (resulting in underdeveloped yeast or off-flavors in aged beer) or too much or too little residual dextrin (which results in an undesirable result of sweeter-than-expected beer) (Kreis and Shewry, 1992). Kilning also influences the rate of lipid oxidation in malt. Lipid oxidation causes the formation of detrimental characteristics in barley and beer. For example, the development of trans-2-nonenal (cardboard off-flavor/aroma) is due to lipid oxidation in malt (Ostergaard et al., 2004; Skylas et al., 2005). For this reason, it is imperative to know the composition of barley when malting.

## The current goal of barley breeding is to optimize both agronomic performance and malting quality

Malted barley (malt) varieties are bred specifically for characteristics that promote good malting and brewing performance. Malt is the primary source in beer that provides starch and enzymes necessary to produce the fermentable sugars which yeast convert to ethanol and carbon dioxide (Bettenhausen et al., 2018). Malt provides not only food for yeast but also color (e.g., melanoidins) and flavor compounds (e.g., esters and aldehydes), which contribute to the final product. There are several parameters that affect the acceptability of malted barley, and it is imperative that malting quality parameters be understood as to their limitations and utilization. These criteria and the preservation of quality control of malt is necessary to ensure consistency, processing efficiency, and quality of the final product in the malthouse, brewery, and distillery (Chi et al., 2003; Anttonen et al., 2010; Wang et al., 2015). The malt Certificate of Analysis (COA) lists the results of these standardized tests that serve to indicate how malt will perform. The COA includes both parameters’ requirements for the maltster and parameters that are useful to brewers to manage and alter the brewing process. These analyses are performed in a laboratory using a standard laboratory-based mashing regime for the wort analyses. The methods for measuring wort are governed by the three main brewing institutions: The Institute of Brewing and Distilling (IoB), the European Brewing Convention (EBC), and the American Society of Brewing Chemists (ASBC). Each institution suggests its own methods. The IoB analysis involves homogenizing and holding the temperature of the mix at 65.5°C, which reflects the use of infusion mashing of ales. The EBC utilizes the Congress mash, found predominantly in the United States, which involves mashing at 45°C and increasing the temperature to 70°C, mimicking the process used in lager brewing (Cremer et al., 2014; Zhang et al., 2017); this process is similar to the ASBC regime. The limitations to the Congress mash remain that it is not reflective of true brewery conditions. For example, the Congress mash method requires milling grains very fine (0.2mm) in the laboratory for analysis; this is very unlike conditions found in a typical brewhouse, in which grain diameter is often contingent upon the mill and brewhouse—this affects predictability of performance. The COA includes many useful features and parameters:

1. Barley variety and crop year indicators. Differences in barley variety (genetics) and crop year or location (environment) affect malting and brewing processes. Barley varieties exhibit highly different malting qualities and flavor characteristics which will influence other quality parameters, such as enzyme levels, protein levels, and flavor. Crop year or location changes affect malting due to differences in dormancy and hydration (Osama et al., 2021).
2. Malt sieve analysis, which serves to measure the size and any damage of kernels. Kernels should be plump and homogenous and should consist of over 90% of the measured kernels. Kernels and husks should be free of damage, to provide a sufficient filter bed during the mashing portion of the brewing process (Finnie et al., 2011).
3. Friability, which is the ability to be crushed/mealiness of a malt. This serves as an indicator of the level of modification and, when used in tandem with β-glucan, can be used to indicate cytolytic modification (Finnie et al., 2011).
4. Malt moisture, which is impacted by steeping, germination, and kilning. Brewers desire malt with values between 4% and 6% due to storage issues (Finnie et al., 2011).
5. Malt extract measures the amounts of fermentable sugars. It determines the amount of alcohol that can be made from 1 t of grain. The higher the extract level, the more alcohol that can be made from a given amount of grain. Malt extract is measured by malting the grain and measuring the amount of soluble sugar (like glucose and maltose) in wort. The measure is presented as a percentage (dry basis). The desired standard for malt is a minimum malt extract level of 80% (Finnie et al., 2011).
6. Protein (soluble) is the primary indicator of proteolytic action, the breakdown of proteins into peptides and amino acids, during the malting process, and the level of modification (Finnie et al., 2011).
7. Color, which is produced by the same mechanism as Maillard reaction products, is the interaction of reducing sugars and amino acids.
8. Protein (total) is inversely proportional to extract, so brewers generally desire a lower protein malt, being cognizant, however, that protein-derived amino acids (called free amino nitrogen or “FAN,” discussed below) are essential to yeast health. Protein, which is barley variety-dependent, is also related to foam stability and haze (Molina-Cano et al., 2001).
9. β-Glucans, polymers of which are found in the cell walls of the starchy endosperm of barley, are highly viscous and can cause a number of problems in brewing, such as reduced rates of wort separation and beer filtration and also the formation of hazes, gels, and precipitates (Simic et al., 2008). β-Glucan can also be used as an indication of cytolytic modification—how much of the cellular structure of the barley endosperm has been degraded during the malting process to unlock access to the starch granules inside (Simic et al., 2008). The germination of the barley grain during malting results in the activation of many enzymes that convert the starch in barley into simple sugars. Beta-glucans persist into finished beer if β-glucanases are not activated during malting (temperatures over 60°C deactivate β-glucanase) or if the malt is poorly modified during the germination portion of malting (Kerr and Schulz BL, 2022). Starch and protein degradation are essential for a clear wort and a resulting beer that is free of off-flavors or textures created by β-glucans.
10. Diastatic power (DP) is the combination of starch-degrading enzymes such as α-amylase, β-amylase, limit dextrinase, and α-glucosidase. DP impacts fermentability and can be influenced by adjusting the mash temperature. Higher levels of enzymes support increased attenuation (reduced residual saccharides/dextrins) and higher levels of adjunct ingredient addition.
11. α-Amylase is one of the most important enzymes to the mash in the brewing process. It progressively breaks open chains of amylose and amylopectin, which are large polymers, into smaller, more bite-sized units, exposing it further to digestion by β-amylase. Through fermentation, most saccharides are taken up by yeast, with the exception of residual dextrins that are integral to the character of beer in terms of sweetness, body, and mouthfeel.
12. Free amino nitrogen (FAN) is an essential part of brewing and can greatly influence the intended flavor of fresh beer and the flavor stability of the beer over time. During aging, remaining nitrogenous compounds tend to form undesirable flavors in the beer. For example, amino acids, such as L-lysine and L-proline, which are absorbed during fermentation at different rates (L-lysine is absorbed rapidly, and L-proline has little to no absorption), also influence the speed of fermentation. A normal fermentation time is about 72–100 h. A wort that is supplemented with L-lysine can complete fermentation in approximately 48 h with complete absorption of L-lysine but can lead to an increase in vicinal diketone levels (VDK) in wort, leading to higher 2,3-butanedione and diacetyl (butterscotch, buttered popcorn) off-flavors over time. This is due to the rapid fermentation time that does not allow the VDK to be reabsorbed by yeast (Picariello et al., 2015). FAN is provided by the malt, and yeast utilizes nitrogen-containing compounds to form enzyme and growth proteins. Wort with high FAN tends to produce excess higher alcohols (fusel) and esters that lead to undesirable flavor. After fermentation, excess nitrogen sources can produce off-flavors in the beer due to increased esters, aldehydes, or fusel alcohol. FAN levels are regulated by monitoring changes in the grist (grain that has been milled in preparation for the mash step of brewing) composition and seasonal variations of raw materials (i.e., barley), to control yeast cultures and overall growth. Most brewing yeast requires approximately 100 mg of FAN per liter of extract for adjunct brewing and 200 mg/l for all-malt brewing to successfully ferment the wort (Kreis and Shewry, 1992; Zhang et al., 2017). Traditionally, large-scale adjunct brewers have sought out barley that is higher in FAN. FAN is therefore important for yeast nutrition—a wort that is lacking in amines can result in a “stuck fermentation” (fermentation that has halted), whereupon the yeast has run out of a nitrogen source and cannot continue to ferment. The resulting beer suffers raised VDK levels (described earlier) leading to increased production of diacetyl, and the unutilized amines that remain in the beer result in a chill haze (undesirable in most beers). Excess FAN can produce off-flavors such as meaty, hotdog, and umami flavors, as well as reduce the stability of the flavor over time (Picariello et al., 2015).

## Starch

All grains and pulses have starch in the form of two polymers, amylopectin and amylose. These long and branched polymers are made up entirely of glucose molecules. These polymers exist in thousands of individual starch granules in hundreds of endosperm cells (compartments) within the endosperm. These polymers will be hydrolyzed by enzymes to single glucose, maltose, maltotriose, maltotetrose, and higher-order oligosaccharides during the malting and mashing stages. During fermentation, yeast will assimilate the glucose, maltose, and maltotriose to produce ethanol. The remaining oligosaccharides will remain in the beer and become part of the metabolome profile. As starch is the most abundant component in grains, then the non-fermentable oligosaccharides will be the most abundant single component in the finished beer (Yu et al., 2019).

The development of starch is relatively conserved for grains and pulses. Starch synthesis genes produce the amylopectin and amylose. There are at least 40 different main genes and minor regulatory genes. Many of these remain in the harvested seed and are more easily identified in the proteome (Finnie et al., 2011). Starch synthase I, II, or III, starch branching and starch debranching enzymes for the development of amylopectin, and granule-bound starch synthase for amylose have been identified in studies in cereals like barley, wheat, rice, corn, sorghum, millet, and buckwheat (Denyer et al., 1994; Smith et al., 1997).

There is some variation in the structure of amylopectin and amylose between the different cereals (Kossmann and Lloyd, 2000; Burton et al., 2002; Wang et al., 2015; Yu et al., 2018). The main function for the starch would be to serve as a source of carbohydrate (glucose) for the growing embryo when the grain is germinating in the soil. Thus, all cereal types have these polymers, but the difference between cereals would be the structure of the granules. These different granule shapes and size and the structure of amylopectin and amylose in these granules will impact the temperature at which the granules become soluble in the brewing process (Tester and Morrison, 1992; Mua and Jackson, 1995; Chi et al., 2003; Zhang et al., 2017). These gelatinization temperature differences could impact the rate at which starch-degrading enzymes start to hydrolyze the starch polymers, especially if the gelatinization temperature is above the temperatures used in the brewing process (Yu et al., 2017; Yu et al., 2018; Yu et al., 2019). This temperature and gelatinization process can impact the rate of starch-degrading enzyme action. Grains like sorghum, corn, or rice have significant higher gelatinization temperatures (peak greater than 65°C) than what is typically used in barley mashing (Chi et al., 2003). Thus, the mashing conditions are set to get optimal starch-degrading enzyme activity as the starch commences gelatinization, or when solid adjuncts such as rice are used; these are boiled before being added to the mash, to allow the malt starch-degrading enzymes to act on the adjunct starch.

The majority of the fermentable sugars, namely, glucose, maltose, and maltotriose, are assimilated by yeast during fermentation. However, depending upon the fermentation conditions, there may be residual amounts of these in finished beer. The non-fermentable oligosaccharides, with four or more glucose molecules, are also found in finished beer. The residual fermentable sugars and all non-fermentable sugars would be classed as metabolites in finished beer (Bettenhausen et al., 2018).

## Protein (so many proteins)

As the next generation of seeds needs a source of energy for growth, *via* the hydrolysis of starch, they will also need a source of protein. This is from amino acids, hydrolyzed from endosperm proteins. The endosperm has numerous protein groups, but the major group is the storage protein, classes as prolamines (abundant in proline and glutamine) (Kreis and Shewry, 1992). While there are hundreds of individual proteins making up to the total amount of protein in grains (Ostergaard et al., 2004; Skylas et al., 2005; Anttonen et al., 2010; Cremer et al., 2014; Osama et al., 2021), the storage proteins alone can account for up to 50% of total amount of proteins (Molina-Cano et al., 2001; Simic et al., 2008). This is because these storage proteins are surrounding the starch granules in every endosperm cell. In the typical malting grains such as barley and wheat, hordeins (barley) and gliadins (wheat) and larger molecular weight proteins glutelin (barley) and glutenin (wheat) make a significant positive contribution to beer foam properties. Similar proteins such as kafirin (sorghum) (Kerr and Schulz BL, 2022) or glutenin (wheat) (Picariello et al., 2015) also show in significant abundance in beers produced from those grains.

In an odd evolutionary step, a major individual starch-degrading enzyme is bound up in the storage protein matrix. Beta-amylase cleaves maltose from the reducing end of starch chains. Maltose being one of the fermentable sugars required in fermentation, the amount and activity of beta-amylase is important for maltsters and brewers. Beta-amylase remains active during malting and the early stages of the brewing process, but it is denatured in final high-temperature (>65°C) stages of the mashing process (Evans et al., 2003). It remains present in the remainder of the brewing and fermentation processes. It has been shown to be one of the most abundant proteins when analyzing beer using proteomics (Jin et al., 2013).

In addition, other proteins such as lipid transfer protein and serpin Z4 and serpin Z7 are reported in high abundance across different grains (Brandt et al., 1990; Asero et al., 2000). A common evolutionary purpose of these is the movement of metabolites such as amino acids, fatty acids, and sugars from the degraded endosperm to the developing embryo. However, these two specific proteins are also important in beer quality, with LTP and serpin Z4 important for foam stability (Evans and Hejgaard, 1999).

During the malting process, around 40 endoproteases are synthesized to degrade the storage proteins (Jones and Lookhart, 2005). Thus, in proteomics, many of these enzymes show in varying abundances, corresponding to a decrease in the abundance of some of the prolamin groups (Kerr et al., 2019; Osama et al., 2021). The use of proteomics is one way to track the decrease in the endosperm storage proteins, due to the increase in endoproteases. Previously, specific enzyme assays for individual endoproteases were very difficult. However, proteomics provides a means to observe differences in abundance between the grain storage proteins and finished malt storage proteins and the expression of the endoproteases.

In brewing, most of these endosperm proteins will continue to show in abundance in finished beer. However, differences in the level of malt modification and brewing process will change the relative abundance of some of these.

As mentioned, amino acids are released by the degradation of storage proteins by carboxypeptidases (Agu and Palmer, 2000). These are quite thermostable and will still be active after the kilning process as well as mashing (Jones, 2005). The amino acids released during germination would typically be used to build enzymes and other proteins during the germination process. However, from the reduced germination time in malting, the amino acids would have limited time to be used by the growing embryo, compared with the longer germination time when the seed is growing in the ground. Further protein hydrolysis during brewing can release more amino acids. The amino acids released during malting and brewing become the important source of nitrogenous energy for a different organism, yeast. The yeast assimilates all the amino acids, except proline. During the fermentation process, after about 4 days, the yeast will start to synthesize its own amino acids, thus becoming self-sufficient. Thus, there are varying amounts of amino acids in beer. In addition, excess production of the amino acids by the yeast can be excreted by the yeast into the beer. These will be identified in as metabolomics analysis of beer.

However, there is more to the uptake of amino acids by yeast. During fermentation, the amino acids are involved in several different metabolic processes, including the production of esters, vicinal diketones (diacetyl and 2,3 pentanedione), and higher alcohols. Many of these are identified in finished beer. The levels of these will vary based on the type of beer being produced and yeast type.

## Non-starch polysaccharides

Cereals are higher in the non-starch polysaccharides, beta-glucan (BG), and arabinoxylan (AX). These make up the aleurone and endosperm cell walls, along with phenolic acids—ferulic acid and acetic acid—with some proteins (Kanauchi et al., 2011). The structure of the endosperm cell walls varies between grain types with barley and oats very high in BG compared with AX (Collins et al., 2010). However, there are smaller amounts of beta-glucan in grains such as wheat (*Triticum aestivum*), rye (*Secale cereale*), sorghum (*Sorghum bicolor*), and rice (*Oryza sativa*), but increased AX, a source of dietary soluble fiber (Dynkowska et al., 2015; Casas et al., 2019).

BG, by comparison, are polymers composed entirely of glucose molecules, but the bonding between the glucose molecules differs compared with the amylose and amylopectin polymers. Regardless, the hydrolysis (cytolysis) of BG during germination of grains can contribute some glucose but mostly longer-chain oligosaccharides (Martin and Bamforth, 1980).

The AX polymer is composed of an arabinose backbone with xylose residues at every three or four arabinose molecules. Enzymes hydrolyze AX during germination, and possibly the mashing stage in brewing would contribute arabinose and xylose to the wort stream (Izydorczyk and Biliaderis, 1995). These are not taken up by the yeast and hence would appear in the final beer (Scheffler and Bamforth, 2005).

## Polyphenols

Barley (and hops) contains significant quantities of phenolic materials (Hartley et al., 1990). Quantitatively of more significance in cell walls are the polyphenols, which have a very complex chemistry. Polyphenols such as catechin and epicatechin can enter into oxidation–reduction reactions. Their ability to be oxidized imparts their classification as antioxidants; however, when they are oxidized, they tend to polymerize and cross-react to form precipitates with proteins. If this occurs in beer, then the result is a haze.

One interesting group of polyphenols is the anthocyanins, which are responsible for the color pigments in the aleurone layers. While most modern barley has no pigmentation, there are forms of barley with blue or black pigments in the aleurone cells. Black barleys are used more commonly in the food industry. Blue aleurone barleys may be used in the brewing industry, but they are not favored as they do not appear as bright compared with barleys with colorless aleurone layers. While these colored barleys are generally considered undesirable for malting, there is no strong evidence showing any negative effect on malting, brewing, or beer quality (Morrissy et al., 2022).

While polyphenols can oxidize in the absence of an enzyme, this process is greatly accelerated by enzymes. Raw barley contains polyphenol oxidase (Clarkson et al., 1992), but this is almost entirely lost during malting. Of much more significance in a mash is peroxidase. This will react polyphenol with hydrogen peroxide (formed by the reaction of thiol groups in gel proteins with oxygen) to form reddish complexes and promote insolubilization. There are a large number of heat-stable isoforms of peroxidase in malt, and their significance has probably been underestimated.

Most grains are a good source of phytosterols (Kaur et al., 2014). Grains contain free and bound forms of phytosterols. Higher levels of phytosterols occur in the outer layers of the kernels. Sitosterol is the most abundant at about 50%, followed by campesterol (15%), with brassicasterol, stigmasterol, δ5-avenasterol, stigmastadienol, and δ7-avenasterol present in varying amounts. It is possible that, if present in very high levels in beer, these sterols could partially serve to inhibit cholesterol uptake.

## Lipids and fatty acids

The lipids are divided into the starch lipids (those associated with the starch granules in the endosperm) and the non-starch lipids (Marion et al., 2003). The amount of lipids varies between different cereal grains, with corn having the highest. Barley contains 75% non-polar lipid (e.g., glycerides), 10% glycolipid, and 15% phospholipid. Perhaps a third of the lipid is present in the embryo, with the remainder being located in the endosperm (starchy endosperm plus aleurone). The lipids are significant because they influence yeast action in fermentation (and therefore beer quality, including flavor) but also because they are detrimental to foam stability and flavor life. A range of fatty acids such as linoleic acid, palmitic acid, oleic acid, linolenic acid, and stearic acid are present and can be level controlled by the growing environment but also malting and brewing conditions as the total lipid content decreases significantly during the malting process as barley is converted into malt and thus into beer. Different barley varieties (and types) contain different lipid concentrations, contents, and patterns (Bravi et al., 2012; Bravi et al., 2017).

Lipids, by definition, have limited solubility in water. For this reason, physical effects are generally more important than chemical or biochemical effects in brewing. The lipids (certainly if undegraded) tend to associate with insoluble components of the mash and therefore are largely lost with the spent grains and become associated with hot and cold breaks.

There are, however, enzymes expressed during malting that are capable of hydrolyzing lipids. The prime enzyme of attack is lipase, which splits fatty acids from glycerol. There is probably a range of lipases that act on different types of lipids. While they are poorly characterized, they are thermostable and retain some activity after kilning and are active in mashing. Wort pH conditions are below the optimal for lipase activity.

Much more attention has been paid to lipoxygenase, which oxidizes unsaturated fatty acids (linoleic acid and linolenic acid) to hydroperoxides. These products can then be converted into staling aldehydes. Barley develops two lipoxygenase enzymes in the embryo. Both enzymes are extremely heat sensitive and are extensively lost during most kilning regimes.

The level of lipoxygenase present in malt declines during storage and may be the reason why malt should be stored for a few weeks prior to use. Lipoxygenase is involved in oxidative reactions that contribute to the formation of the Oberteig layer in the lauter grain bed that can reduce wort runoff speeds, and when the level of the enzyme reduces, the ease of wort separation improves.

## Vitamins and minerals

All cereal grains are a significant source of macro- and micronutrients, containing carbohydrates, proteins, dietary fibers, vitamins, and minerals (Poole et al., 2021). Nutrient proportion varies among different cereal grains and is dependent on processing conditions. In wheat, for example, there is a higher amount of nutrients within the seed coat as opposed to that in the endosperm. When the grain is milled to generate refined flour, this processing reduces the nutrient content (Oghbaei and Prakash, 2016).

Vitamins are essential micronutrients for growth, metabolism, and reproduction. Vitamins are classified into two groups based on solubility: fat-soluble (A, D, E, and K) and water-soluble (B and C) (Jones and Anderson, 2008). As the human body cannot synthesize vitamins (except for D and B1), it is imperative that they are taken in from various outside sources. Cereal grains are a good source of vitamins A, B, and E but are less abundant in other vitamins (Garg et al., 2021). The minerals calcium, magnesium, potassium, phosphorus, iron, and sodium are found in various quantities in cereal grains. Rye, for example, has many health benefits including fiber, protein, iron, magnesium, phosphorus, and zinc. The darker the rye, the more vitamins and minerals it contains, generally speaking.

## Malting

Within a short time after humans discovered the need to have grains germinate to produce a sweeter-flavored water with a novel side effect (i.e., beer), they started specific germination and drying processes. These deliberate and controlled wetting (steeping), germination, and drying are called the malting process (Briggs, 1998).

**Figure 1:**
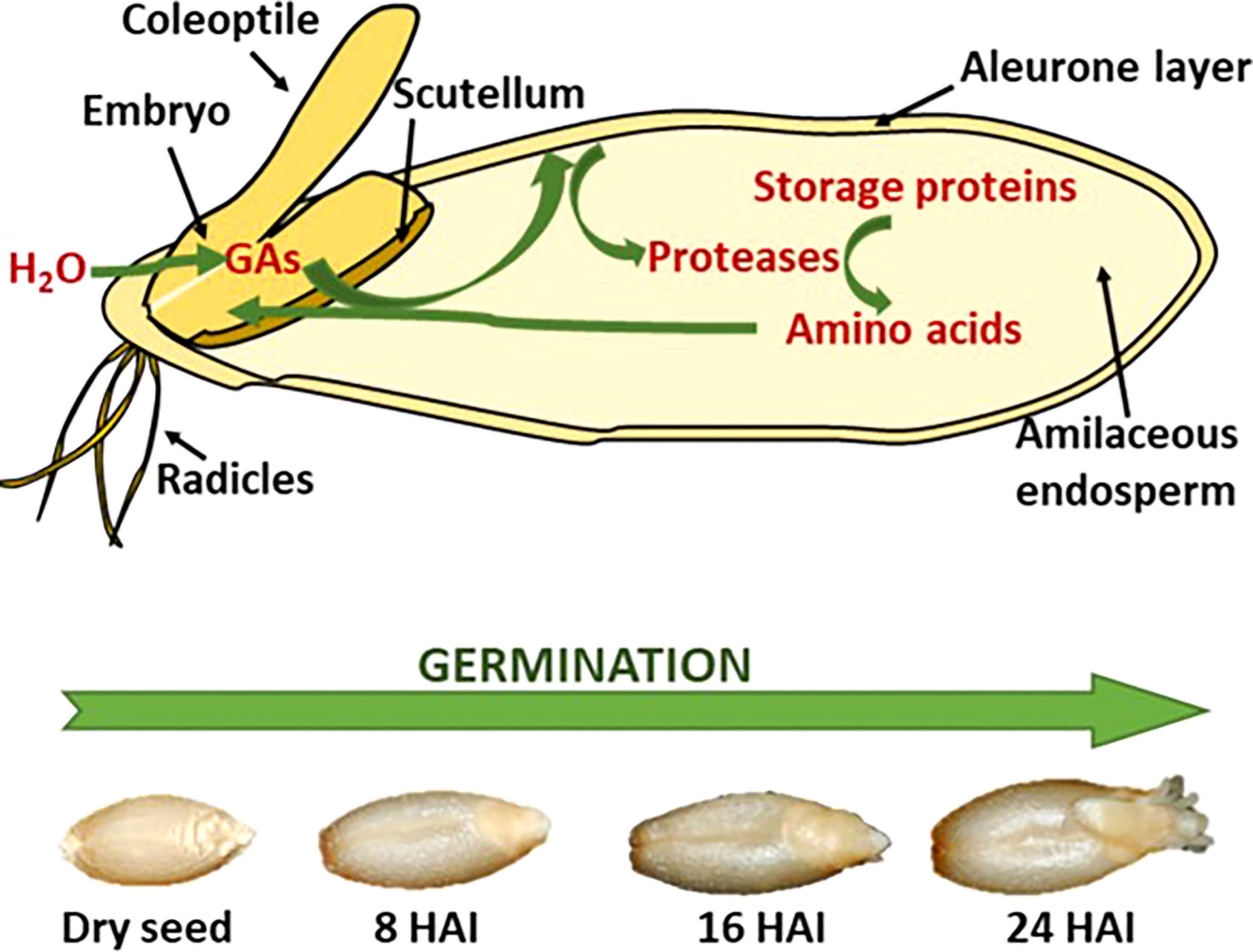
Schematic representation of storage protein hydrolysis in germination cereal seeds **(A)** with the visible changes of barley during the germination process, showing hours after inhibition (HAI) **(B)** (Diaz-Mendoza et al., 2019).

There are three distinct steps in malting, as described above in detail: 1. steep, 2. germination, and 3. kilning. Steeping is the initiation of multiple enzymic pathways where major enzyme groups, non-starch polysaccharides, storage proteins, and starch-degrading enzymes are synthesized. Germination sees the progression of these enzymes from the embryo and aleurone cells into and through the endosperm (Briggs et al., 1981). **Figure 1** shows the progression of synthesized enzymes through the endosperm and the observable changes as the embryo produces an acrospire.

**Figure 2:**
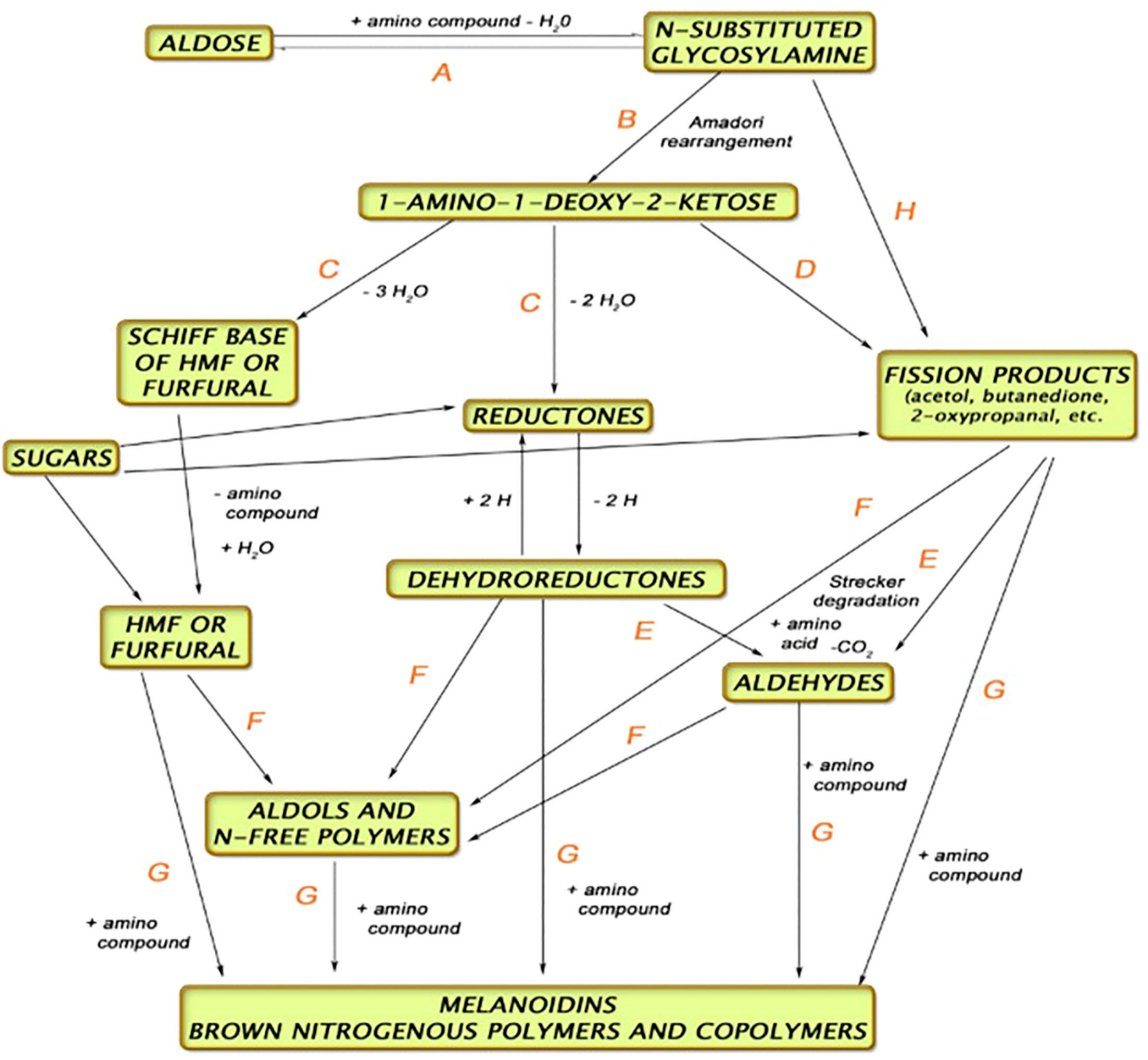
Pathway for Maillard and Strecker reactions during kilning (Anon, 2017).

The kilning process serves several functions, namely, to reduce the water content of the grain, preserve the enzyme activity, and induce color reactions (Maillard and Strecker Degradation compounds—**Figure 2**) (Briggs et al., 1981). Drying the malt to temperatures >90°C will induce more color and flavor compounds. These more colored (specialty) malts are used to produce darker beer styles other than typical lagers and ales.

The changes in the expression of enzymes and loss of the storage proteins can be observed using proteomics (Jin et al., 2013; Picariello et al., 2015; Kerr et al., 2019; Osama et al., 2021). The resultant increase in sugars, amino acids, organic acids, fatty acids, and Maillard and Strecker compounds are observed through metabolomics analysis (Bettenhausen et al., 2018).

Despite the advances in these complex ‘omics technologies, the malting industry is still conservative about the methods used to test quality. Only a handful of key quality factors are measured, including Extract, starch that enzymes extract and convert into fermentable and non-fermentable sugars during the mashing process (of brewing) (Evans et al., 2003; Yu et al., 2019). These sugars act as food for yeast to create alcohol and help create body in beer; enzymes to degrade the starch (alpha amylase and diastatic power); and the total amount of free amino acids (FAN, including all nitrogen sources) (Agu and Palmer, 2000). Unfortunately, these may not be specific enough to provide the best information to brewers about the efficient brewing and flavor that will come from malts. These measurements are only routinely taken on barley and wheat malts. Other grains that are malted, such as rye, millet, and sorghum, have not had routine testing developed around those aforementioned properties.

## Wort properties

Different amounts of malts will be mixed with warm to hot water to continue the hydrolysis of the major endosperm reserves but specifically to break down as much as possible to maltose and glucose. This is the first stage of brewing and is called mashing. Other than some of the enzymic reactions required to increase amino acids and sugars for fermentation which will happen during mashing, all the water-soluble compounds present in the grain along new compounds created during malting, such as Maillard and Strecker reactions, will be dissolved into the water. This liquid matrix of thousands of proteins and metabolites is wort. After the mashing stage, the wort is separated from the malt. This is where the contribution from the grain and malts has been fully reached. The next stage of brewing is boiling where hops are added to the wort for bitterness, and then fermentation, where yeast is added, for alcohol and CO_2_ production.

The fermented beer has some obvious characteristics from the starting grain and malt such as the color and some sweetness, depending upon beer style. The amounts of hops added can quickly overwhelm any aroma and taste derived from the grains or malt. Most metabolomics studies exploring the contribution of the grain and resultant malt contribution to beer typically use light to moderately hopped beer styles with barley as their main source of fermentable sugars and proteins. These studies have allowed grain and malt researchers to better understand the variation in compounds within different functional groups based on the type of grain, the growing region of the grain, or the level of malting. There are few studies based on the study of alternative grains such as oats, rye, and wheat for purposes other than nutrition. Most studies on these grains regard the health aspects, baking properties, and subsequent organoleptic properties (Sánchez-Martín et al., [[NoYear]]; McKee and Latner, 2000; Busko et al., 2010; Belobrajdic and Bird, 2013; Khakimov et al., 2014; Heiniö et al., 2016; Koistinen et al., 2018).

## Specific metabolomics analysis

In the brewing industry, there is a relentless pursuit for quality control, brand development, and customer satisfaction. The quality of all raw materials used in brewing (malt, water, yeast, and hops) and their impact on final product flavor is becoming increasingly important. Simple sensory evaluation of raw materials gives an immediate indication of quality and/or the presence of any off-flavors, disease, or mishandling. Current sensory analysis of malt is not, however, a requirement, nor is it normalized or standardized. Brewers have historically viewed “malt quality” as a perception of personal views which encompass specifications, which they believe protect the integrity of their flagship or brand. However, the “malt spec” is by no means as reliable as many brewers would admit. Congress mash has historically been utilized to evaluate malt quality but has recently been subject to scrutiny and change (Evans et al., 2003). Briefly, the congress mash is a standardized small-scale mashing procedure employed to assess malt quality whereupon the specific gravity of the wort permits calculation of extract. Other analyses which are rendered by the congress mash are odor, turbidity, pH, color (including after boiling), viscosity, soluble nitrogen content, free amino nitrogen, and final degree of attenuation (Jin et al., 2013). There has recently been an incentive to standardize the malt sensory process and the lexicon used. The malt hot steep sensory method, validated by ASBC in 2017, was designed for the industry and by the industry with the intent of doing just this—standardizing the method and the way malt is talked about as it is evaluated (Asero et al., 2000). The hot steep method is, briefly, a hot water extraction of malt, whereupon malt is not treated as if it is to be brewed (as in the Congress Mash) but simply for a sensory assessment of malt quality (Brandt et al., 1990; Evans and Hejgaard, 1999). The data generated by the hot steep method provides information regarding sample quality *via* the aromas and flavors present; in other words, it generates a “fingerprint” for each malt type in terms of aroma and taste characteristics and based on the variety of barley utilized, the environment it was grown in, and how it was malted. One outcome of this method is that undesirable traits or traits that are directly connected to malt or barley imperfections are easily identified. As of the time of this study, it was not possible to correlate malt hot steep sensory with beer flavor. This is considered one of the main product attributes that influences food choice, such as in breads or baked goods. Healthier or alternative food options, such as gluten-free or sugar-free, are often lacking in flavor quality, which obstructs consumption and thus has a measurable health impact. Historically, flavor research has focused on improving flavor by decreasing or masking undesirable attributes or increasing levels of desirable attributes or attributes able to mask other undesirable attributes. The scientific approach to this has been to define the flavor profile, by using a trained sensory panel, then analytically identify the chemistry which drives those attributes. This methodology involves separating out the chemical components by chromatography and tasting or smelling them individually and in isolation to determine which compounds evoke the attributes of interest. While these methods are unquestionably effective at identifying sources of off-flavors, or for finding characteristic flavor compounds, they have limited ability to define sensory interactions or complexity. To advance our understanding of flavor perception, driven by many chemical factors, a holistic approach which considers the sum and the interplay of those factors and their relationship to the sensory attributes of interest is needed. Current studies have revolved around barley and, to a lesser extent, wheat and sorghum, for brewing. More research is needed on grains that are malted and used for brewing in order to understand their relationship to malt quality and sensory.

Metabolomics is an increasingly promising field in the height of development within the food and beverage industry. Metabolomics is the scientific study of chemical processes involving metabolites, which are the small-molecule substrates, intermediates, and products in food. Metabolites are chemical fingerprints that processes (such as malting and brewing) leave behind, and metabolomics is the study of their small-molecule metabolite profiles (called the metabolome).

Metabolomics, generally, is a non-targeted (i.e., assessment of all metabolites at once as opposed to “targeted,” where one is looking for specific metabolites such as amino acids or sugars) approach for comprehensive, qualitative, and quantitative analysis of all metabolites. Multiple platforms and technologies are often used in conjunction for metabolomics-based approaches because each technique has inherent limitations as to the breadth of coverage. Commonly utilized platforms include chromatography coupled with mass spectrometry (MS) or nuclear magnetic resonance (NMR) (i.e., gas chromatography mass spectrometry (GC-MS), liquid chromatography mass spectrometry (LC-MS)). Since the late 2000s, there have been an abundance of studies using metabolomics to profile raw ingredients, beer, and wort. In wort, it is important to understand both the broad view and the narrow view of composition to understand what will happen in beer. Metabolomics can be used to measure plant metabolism in real time, considering both primary and secondary metabolism, for example in the context of plant physiology and a plant’s response to external stresses. An important topic in the brewing industry is how agronomic factors affect the quality of the end product. Variations in growing and processing conditions (e.g., high *vs*. low nitrogen in the field) have an influence on the quality of grain which subsequently may influence malting, beer, and distillate quality (Herb et al., 2017b; Herb et al., 2017a).

**Figure 3:**
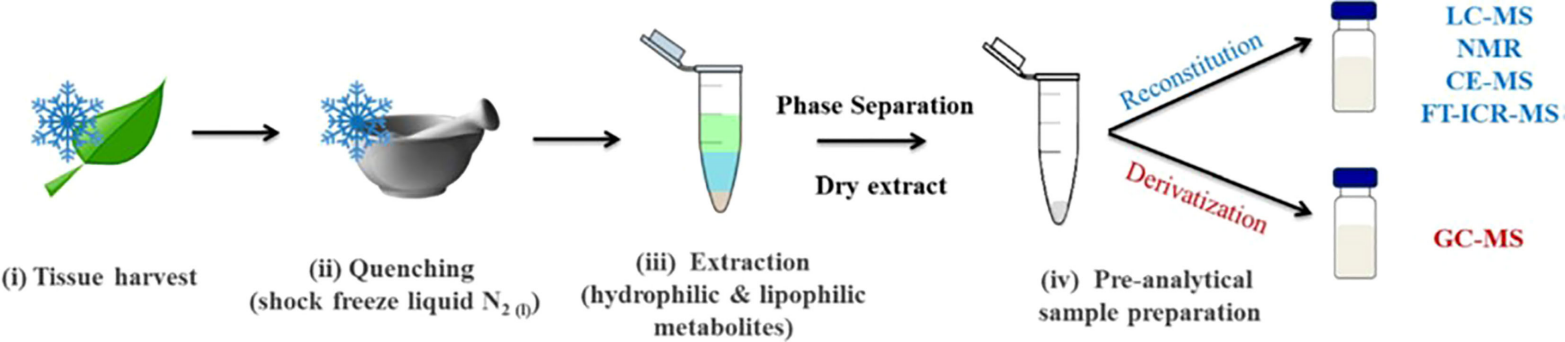
Typical sample preparation workflow used in plant metabolomics studies (Jorge et al., 2016).

Given the recent development of high-throughput sensory techniques and the numerous chemical and genetic factors involved in crop selection, metabolomics is a useful and relevant technique for investigating cereal crop data. Metabolomics allows for the application of large-scale chemo-sensory data collection to the analysis of different crop varieties. This method was applied to barley either alone or in combination with supporting sensory data to make determinations about crop quality. In each case, the barley was tested in the context of its most relevant agricultural product: wheat was tested in the form of bran (since most wheat is consumed in the form of bread), and barley was tested as brewing malt (since most barley intended for human consumption in the United States is used for brewing malt, and a smaller portion as bran). This demonstrates the efficacy of metabolomics techniques as part of the decision tree of quality control for cereal grains; despite their different products and uses, each species benefit from metabolomics analysis. The past 20+ years have been subject to some of the most striking scientific advancements in many different fields due to the development of high-throughput analytical techniques and advanced omics technologies (Capozzi and Bordoni, 2013). The central dogma of molecular biology involves four major “omics”: genomics, transcriptomics, proteomics, and metabolomics. Metabolomics is an increasingly promising field in the height of development within the food and beverage industry. It is the scientific study of chemical processes involving metabolites, which are the small-molecule substrates, intermediates, and products in food. Metabolites are chemical fingerprints that processes (such as malting and brewing) leave behind, and metabolomics is the study of their small-molecule metabolite profiles (called the metabolome) (Schmitt-Kopplin, 2021). **Figure 3** shows the steps from biological sample to metabolite separation and identification. In the case of malting and brewing, the starting sample could be the grain, processed malt, or finished beer.

Metabolomics, generally, is a non-targeted (i.e., assessment of all metabolites at once as opposed to “targeted,” where one is looking for specific metabolites) approach for comprehensive, qualitative, and quantitative analysis of all metabolites (Sumner et al., 2007). Multiple platforms and technologies are often used in conjunction for metabolomics-based approaches because each technique has inherent limitations as to the breadth of coverage. Commonly utilized platforms include chromatography coupled with mass spectrometry (MS) or nuclear magnetic resonance (NMR) (i.e., gas chromatography mass spectrometry (GC-MS), liquid chromatography mass spectrometry (LC-MS), ultraviolet-visible (UV-vis) spectroscopy, and near-infrared (NIR) spectroscopy and more (Cavallini et al., 2021).

A novel branch of metabolomics works to characterize and profile food metabolites and is able to describe assessments of quality, authenticity, safety, and traceability (Capozzi and Bordoni, 2013). The metabolome of food (and beverages) represents the collection of small molecules (molecular weight <2 kDa) called metabolites that are present in foods and are derived from animals, plants, and microorganisms. Metabolites in foods such as barley, malt, and beer are subject to changes during processing (such as malting, brewing, fermentation), storage, aging, microbial decomposition, and dynamic chemical reactions or contamination. Each variety of barley, batch of malt, and glass of beer has its own characteristics depending upon the presence and abundance of certain metabolites and the combination thereof (Bettenhausen et al., 2018). Herein lies the usefulness of metabolomics in understanding the connections between genotype, environment, and phenotype (*via* sensory or quality assessments). The chemistry of barley influences brewing parameters and the final beer quality. Recent studies have demonstrated that small molecules in barley grain are highly variable among barley genotypes and that interactions between barley genetics and environment (G×E) further influence the chemistry of the malt (Beal, 1994; Frank et al., 2011). Malting quality is based on both differences in protein structures of barley (which tells us there is variation in enzymatic action) and the expression of the genes involved (Heuberger et al., 2014). The metabolomics approach allows us to understand the mechanisms and indicators of certain biochemical processes, such as determining the compounds responsible for certain flavor traits in beer and the routes that these compounds take to arrive at the point where a staling/off-flavor is created (Hughey et al., 2015; Spevacek et al., 2016; Bettenhausen et al., 2020).

**Figure 4:**
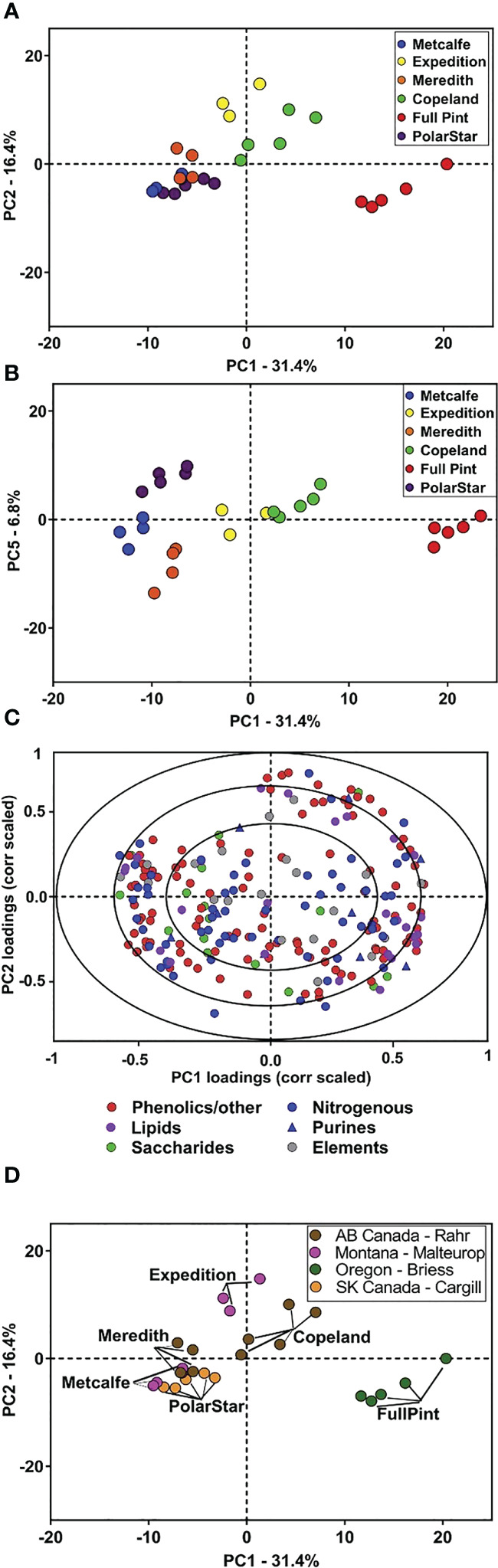
Principal component analysis (PCA) of malt metabolites of the six malt sources. PCA was performed on the 217 annotated malt metabolites for the six malts. **(A)** PC score plot for PC1 and PC2 of six malts. **(B)** PC score plot for PC1 and PC5 provides additional separation among the malt sources. **(C)** Correlation-scaled loading plot for PC1 and PC2 of the six malts, colored according to chemical class. **(D)** PC score plot (PC1 vs. PC2) colored according to each of the four maltsters. Analyses were based on n = 3–5 extraction replicates per source (Bettenhausen et al., 2018).

Metabolomics studies have recently been utilized in investigations to identify new markers of quality traits to breed superior plant lines (Heuberger et al., 2014; Khakimov et al., 2014). The results of this study suggest that metabolism and quality traits are co-influenced by barley G×E factors and demonstrate the usefulness of metabolites as efficient markers of quality traits, suggesting the need for further research into the metabolites and biochemical processes which may contribute to beer flavor and flavor stability. Given the breadth of variation in malt metabolites due to barley genotype described, there is the potential that barley genotype may also influence the flavor stability of beer. Malt plays a key role as the keeper and transporter of precursors for many of the flavor compounds in beer. Previous research demonstrates that barley genetics can influence beer flavor. However, the chemical basis for differences in beer flavor attributed to barley variety is only in the beginning stages of definition (Dong et al., 2015; Herb et al., 2017b; Bettenhausen et al., 2018; Bettenhausen et al., 2020; Windes et al., 2020). Broadly, there are several chemical classes of interest regarding malting and brewing which continue to be investigated by studies involving metabolomics. **Figure 4** provides an example of identifying metabolites from different barley varieties, then key metabolite groups based on varietal distinction or malting process.

## Malt metabolite chemistry: role of sulfur compounds

Sulfur compounds in malting and brewing are of utmost importance. These compounds are volatile, with very low organoleptic thresholds (detectable in parts per billion). Trained sensory panels can recognize sulfur attributes even when the compounds are undetectable by analytical instruments. Sulfur compounds, to complicate the issue, are sensitive to changes in pH and temperature and thereby readily convert into other compounds in response to those changes. Sulfur compounds which react with ketones create a catty or barnyard goat-like aroma in beer (Vermeulen et al., 2006; McGorrin, 2011; Martins et al., 2020). Sulfur flavor is desirable in some styles of beer (e.g., Saison Farmhouse style) but is generally associated with aging and poor flavor stability. For example, 3-methyl-2-butene-1-thiol (associated with “lightstruck” flavor), which is often found in beer exposed to excessive light or aging and is caused by a reaction between hop α-acids and riboflavin in beer, is easily controlled by proper quality control and monitoring of the compounds. Sulfitic attributes, derived from sulfuric acid or sulfate salts (SO_4_^-)^, include sulfur dioxide (lit match) and hydrogen sulfide (rotten eggs). Sulfidic (hydrogen sulfide, H_2_S) attributes comprise mercaptans (garbage in methyl mercaptans, whereas higher mercaptans give off burnt rubber), *garlic, light struck* (*skunky*), autolyzed yeast (*hot dog water*), and butyl mercaptans (*shrimp-like*). Vegetative sulfur attributes include dimethyl sulfide (DMS) and dialkyl sulfides, which give off cooked/canned vegetable properties. Whereas sulfitic attributes are rare and regulated (legally restricted to 10 ppm) in the U.S., sulfidic flavors, produced naturally, are not regulated, but they are considered very undesirable in beer. Hydrogen sulfide, thiols, and mercaptans are being widely studied regarding their control, origin, and biotransformation during malting and brewing (Vermeulen et al., 2006; McGorrin, 2011; Belda et al., 2016; Ferreira and Guido, 2018).

## Malt metabolite chemistry: role of lipids

Although not a major component of beer, free fatty acids (FFA) are considered undesirable in the finished product. FFAs are a “foam-negative” compound, relating to their surface absorption tendencies upon interaction with foam-positive proteins (Bamforth, 1993; Spevacek et al., 2016). Medium-chain fatty acids such as hexanoic, octanoic, and decanoic acid can result in off-flavors as *rancid, vomit, goat-like, cheese-like*. These volatile off-flavors are formed by the yeast during fermentation (Castellari, 2001; Nardini, 2004; Horak et al., 2009). However, long-chain unsaturated fatty acids, such as linoleic and linolenic acids, are more often derived from malt and lead to the formation of staling off-flavors (lipid oxidation) in beer. Saturated fatty acids (e.g., palmitic and stearic) from malt are also related to gushing (spontaneous foaming over when beer is opened). Trans-2-nonenal, a common staling compound in beer, is formed from oxidized lipid components. Lipid oxidation in malt has also been reported to cause lautering (filtering) problems during the brewing process and contribute to some downstream off-flavors (Kaukovirta-Norja et al., 1998; Su et al., 2022). In recent years, an effort to create low-lipoxygenase malt (LOX-less) has been made. The kilning process affects the lipoxygenase in malt and its ability to oxidize lipids. Fatty acids in barley and malt also play a role in the amount of extract obtained from brewing and the attenuation (the percentage of sugars converted into alcohol and carbon dioxide by fermentation) (Cozzolino et al., 2015).

## Malt metabolite chemistry: role of amino acids and other nitrogenous compounds

Amino acids, from a malting and brewing perspective, are of utmost importance to understand. They are the most readily available source of nitrogen for yeast, derived from malt and wort, and they are critical to fermentation performance and beer quality. Amino acids partially make up FAN, which is defined as the sum of individual amino acids, ammonium ions, and small peptides in wort (Lekkas et al., 2005). All-malt wort typically contains ~19 amino acids, which are taken up preferentially by yeast (Bettenhausen et al., 2018; Ferreira and Guido, 2018). The uptake of amino acids and creation of by-products of yeast create changes in wort composition which influence organoleptic properties of the finished product. Changes in the concentrations of amino acids in wort tend to influence and change nitrogen metabolism because the yeast amino acids are derived from the amino acids in the wort (Carvalho et al., 2015; Ferreira and Guido, 2018). However, amino acid concentration has been shown to vary from 100 to 5,000 mg/l depending on beer style and barley variety used (Yin and Aron, 2018). The most important amino acids in wort are isoleucine, valine, phenylalanine, glycine, alanine, tyrosine, lysine, histidine, arginine, and leucine. These amino acids are responsible for the “regulation of biosynthesis of flavor-active compounds formed by yeast” (Ferreira and Guido, 2018).

## Malt metabolite chemistry: Maillard reaction products

Malt is the most reliable source for Maillard reaction products (MRPs), but beer is complex and the exact origin of MRPs created in beer is still under investigation (Hellwig and Henle, 2020). Applying metabolomics methods, such as high-pressure liquid chromatography (HPLC) combined with ultraviolet detection (UV), has proved to be a successful venture into identifying the etiology and pathway of MRPs in brewing. It has been shown that MRPs which are created in malt, through hot steep extraction, do not correlate with what is in beer (ASBC, 2017; Bettenhausen et al., 2021), and so it is important to understand the transformation during brewing. Maillard reactions occur more commonly at higher temperatures, with low moisture levels, and under basic conditions with pentose sugars (e.g., arabinose, xylose) rather than with glucose and maltose (hexose, disaccharides) and with amino acids that have more propensity to react, such as lysine and glycine. Melanoidins, a result of the Maillard reaction, lends to color in malt and beer. Maillard reactions occur both during the kilning stage of malting and in the kettle during the wort boiling stage of brewing (Martins et al., 2000).

There are three stages in the creation of MRPs. The first is the creation of Amadori rearrangement products (ARP) through the reaction of amino and imino groups in free amino acids, peptides, and proteins with reducing sugars. The most common ARPs are N-ϵ-fructosyllysine (FL) and N-ϵ-maltulosyllysine (ML). Both compounds have been identified in malt and beer. The second stage is the degradation of ARPs into vicinal dicarbonyl compounds (e.g., glyoxal, 3-deoxyglucosone) and the formation of dicarbonyl compounds in a reaction with polysaccharides—this is what is known as “caramelization,” and many compounds are formed as a result of this, such as furfural (Hellwig and Henle, 2020), from the dehydration of the aforementioned ARPs. The third stage involves formation of glycated amino acids and Strecker degradation, which forms aldehydes present in beer during fermentation. There are many MRPs yet to be investigated in malt and beer, as well as understanding how processing, barley variety, malt type, and the interaction with other raw ingredients (hops, yeast) may affect quality and flavor of malt and beer regarding those products.

**Figure 5:**
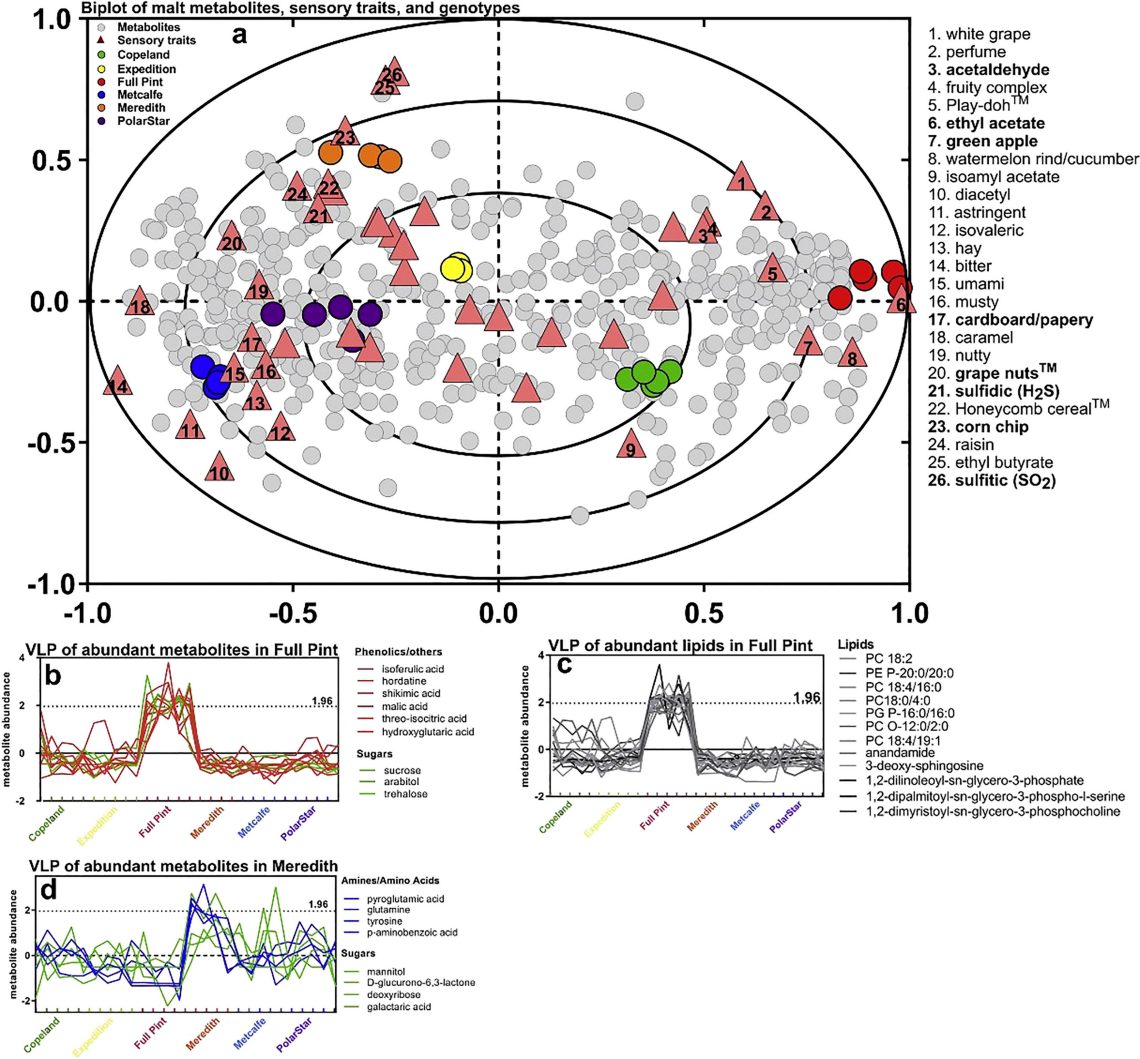
Multivariate association of malt metabolites with beer sensory traits after 2 months of storage. The association between beer metabolites and beer sensory was evaluated with two-way orthogonal partial least squares (O2PLS) and performed on 217 metabolites, 20 metals, and 45 sensory traits: **(A)** O2PLS overview biplot of all 217 malt metabolites and 20 metals, source, and sensory trait associations showed separation among the six sources and co-variation with sensory traits and metabolites. O2PLS score and loading plot of the metabolites/metals (gray circles), sensory traits (triangles), and malt sources (circles). Sensory traits associated specifically with Meredith or Full Pint are denoted as bold text. Numbers in triangles correspond to sensory traits (right of plot). Variable line plots (VLP) graphed the contribution of more highly abundant metabolites contributing to sensory traits associated with each beer source. **(B)** VLP of malt metabolites (non-lipids) most abundant in Full Pint, as explained by the O2PLS model that includes all metabolites and metals. **(C)** VLP of malt lipids most abundant in Full Pint. **(D)** Variable line plot of beer metabolites most abundant in Meredith, as explained by the O2PLS model that includes all metabolites and metals. z of 1.96 approximates 95% confidence that a metabolite is higher in a sample (Bettenhausen et al., 2018).

Even with a large number of metabolites, variation in concentrations of these may not be detected in any sensorial process. **Figure 5** shows variation in metabolite profiles that are associated with sensorial properties in beer. These sensorial properties can change depending upon beer styles and the age of the beer. Metabolomics allows researchers to detect specific metabolites that may change on processing or storage conditions, which could impact taste and or aroma.

## Broadly, chemical variation can result in diversified breeding

The understanding of chemical variation among malting barley varieties will allow for more diversity in breeding programs for flavor development/improvement. Fermentation and processing studies (for beer and distilled spirits) have advanced our knowledge of quality control for the raw ingredients in these products. Although the available body of knowledge seems vast, there is much to be learned about the complex nature of fermentation and production of wares such as beer, distilled spirits, breads, and other value-added manufactured goods. These products have multiple raw ingredients, processing steps, and complex chemistry; the result is that multiple factors influence their sensory quality. As such, an equally complex method of analysis is required to examine the relationship between quality and chemosensory properties of barley. Similarly, although barley is one of the world’s essential cereal crops, its quality as a foodstuff is not well-matched with known organoleptic properties. The value of metabolomics to assist in the selection of varieties for malting (brewing) is still being assessed as malting barley is a chemically complex crop and the same compounds that affect its functionality for food/beverage preparation also have bearing on its flavor. Breeding for malting quality barley is an extended time- and effort-involved process. Breeding programs have excelled at choosing potential malting cultivars for yield, disease resistance, and, to a lesser degree, malting quality. However, this selection has also led to a narrowing of the genetic pool resulting in a decrease of genetic diversity and flavor traits (e.g., as described in tomato) (Bartoshuk Linda and Klee Harry, 2013; Cuesta-Marcos et al., 2016). In the world of brewing, hops have variety names (e.g., Cascade, Centennial), whereas malt is more commonly referred to the stylized product of the malting process (e.g., two-row, base, Munich), and not by the barley from which it originated. This disparity has developed within the brewing industry because barley is utilized not as a plant but as a processed seed (malt). These grains have been domesticated and utilized for thousands of years, and humans have created thousands of varieties of barley. However, over time and through much culling of agriculturally undesirable barley (according to “big” beer), brewers currently rely on a short list of acceptable malting and brewing varieties (AMBA, 2020) governed by the aforementioned AMBA. This to “help ensure an adequate supply of high quality malting barley for the malting, brewing, distilling, and food industries” (AMBA, 2017; AMBA, 2020). This practice, however, has narrowed the genetic diversity of barley, creating a genetic bottleneck of varieties able to perform agronomically, but unable to provide novel malting quality and flavor traits. The governance of barley research and limited variety releases has resulted in barleys bred for large adjunct brewers (such as Coors and Anheuser-Busch InBev). Traditionally, these large-scale adjunct and high-gravity brewers have required barleys with traits such as high diastatic power levels, capable of extraordinary enzymatic effort to convert adjunct sugars. What is needed, regarding diversity, is barleys bred for all-malt beers brewed in the U.S. Large maltsters require barley that is consistent and efficient; diversity is admonished due to the technical challenges it presents for automation and throughput. Heirloom barley varieties may offer the U.S. market the opportunity for craft to build a new market for novel varieties, which lend agronomic, quality, and flavor diversity and a new tool for craft brewers to use in the creation of unique products. Because malting chemistry is so specific, there is correspondingly narrow genetic diversity within cultivated malting barley varieties. Only 34 two-row and 7 six-row malting varieties were approved to be grown in the 2023 season in North America by AMBA. This is a conundrum which has imposed unintentional restrictions on flavor and product differentiation in beer, as recent work shows that barley genetics influence beer flavor. As such, breeding programs have begun to investigate the introduction of heirloom cultivars for the purpose of exposing domesticated varieties to new genetics. Metabolomics is well-positioned to predict how these varieties may influence both the chemistry of the malt and the flavor of the resulting beer. Other varieties do not have the same carefully crafted parameters for malting and brewing, which limits the growth of quality and de-commoditization (Brouwer et al., 2016).

## Metabolomics approaches combine well with supporting sensory methodology when appropriate

Over the past few decades, evaluating wheat and barley, malt, and beer using untargeted methodologies has successfully identified hundreds of chemical compounds responsible for organoleptic properties such as sensation (mouthfeel, such as tingling from carbonation) and aroma. Several compounds identified in the literature have exhibited good correlation with specific sensory descriptors and have been used as chemical markers for attributes. Still, evaluation of barley malt based on taste or aroma perception separately is rather limiting. Malt and beer should be evaluated in their original matrix, where all human senses can integrate to achieve a perception of the product as a whole. In terms of malt flavor quality, there is lack of chemical and sensorial agreement which has created inconsistency and ambiguity in the term “quality” in literature. Despite many extensive studies, the key to highly desirable or undesirable characteristics of malt and beer is still not fully understood. The difficulty in capturing a holistic sense of the word “quality” can be attributed to the complexity of malt and beer matrices in the analytical and sensory aspects. Many papers lack experimental design that includes comprehensive chemical and sensory evaluation. The causative relationship between the chemistry and the quality of both wheat and malt/beer has only recently been studied.

Studies on relationships between malt and beer sensory and chemistry has just recently begun. Malt’s contribution to the final profile of beer is complemented by hops and yeast fermentation by-products, creating a unique organoleptic experience. However, much of the flavor contributed by barley has been attributed to the malting process and thus breeding programs have focused on malt quality outcomes, rather than positive or unique flavor attributes. The current range of malts available to brewers, which are pale or base malts, have not been considered major contributors to a beer’s overall “malt flavor” profile. Craft brewers have recently shifted focus, asking barley breeders and maltsters to provide malting quality specifications better suited for all-malt brewing, including lower protein, DP, and FAN (BA, 2014; Shrestha and Lindsey, 2019). Craft brewers have also continued to demand commercial “heirloom” varieties (e.g., Barke^®^, Golden Promise^®^, and Maris Otter^®^) based on anecdotal contributions to beer flavor, despite the fact that these varieties do not adhere to traditional agronomic and malting quality standards (Windes et al., 2020). Developing a deeper understanding of the contributions of barley genotype to malt and beer flavor is an area of active research (Herb et al., 2017b; Herb et al., 2017a; Bettenhausen et al., 2020; Windes et al., 2020).

It has been demonstrated that commercially available malts of similar type—but made from different genotypes and malted at different locations—had different metabolite profiles and led to different beer sensory outcomes. Metabolomics is a powerful tool for the evaluation of malt and beer flavor because it is able to identify volatile compounds and precursor compounds using various types of chromatography and mass spectrometry (Heuberger et al., 2014; Bettenhausen et al., 2018; Diez-Simon et al., 2019; Bettenhausen et al., 2020).

## Protein composition *vs*. protein content

Typically, grains are tested for protein content post-harvest. The protein content would indicate an acceptable level of protein for a specific process. However, the composition of the protein is not really considered. For protein composition, we need to explore the hundreds of individual protein types. This is beyond the electrophoresis or liquid chromatography separation. Gel electrophoresis separating on size and then isoelectric focusing separating on charge was the first was to identify the hundreds of individual proteins (Skylas et al., 2005; Bak-Jensen et al., 2007; Finnie and Svensson, 2009).

The latest methodology for proteomics uses specific enzymes (proteases) to cut up all proteins into peptides in the samples whether these are a grain, malt, wort or beer samples (Colgrave et al., 2013; Picariello et al., 2015; Schulz et al., 2018; Kerr et al., 2019; Marcus et al., 2022). The resultant peptides are separated in a liquid chromatography system and then identified by mass spectroscopy databases containing peptide sequences from known species.

**Figure 6:**
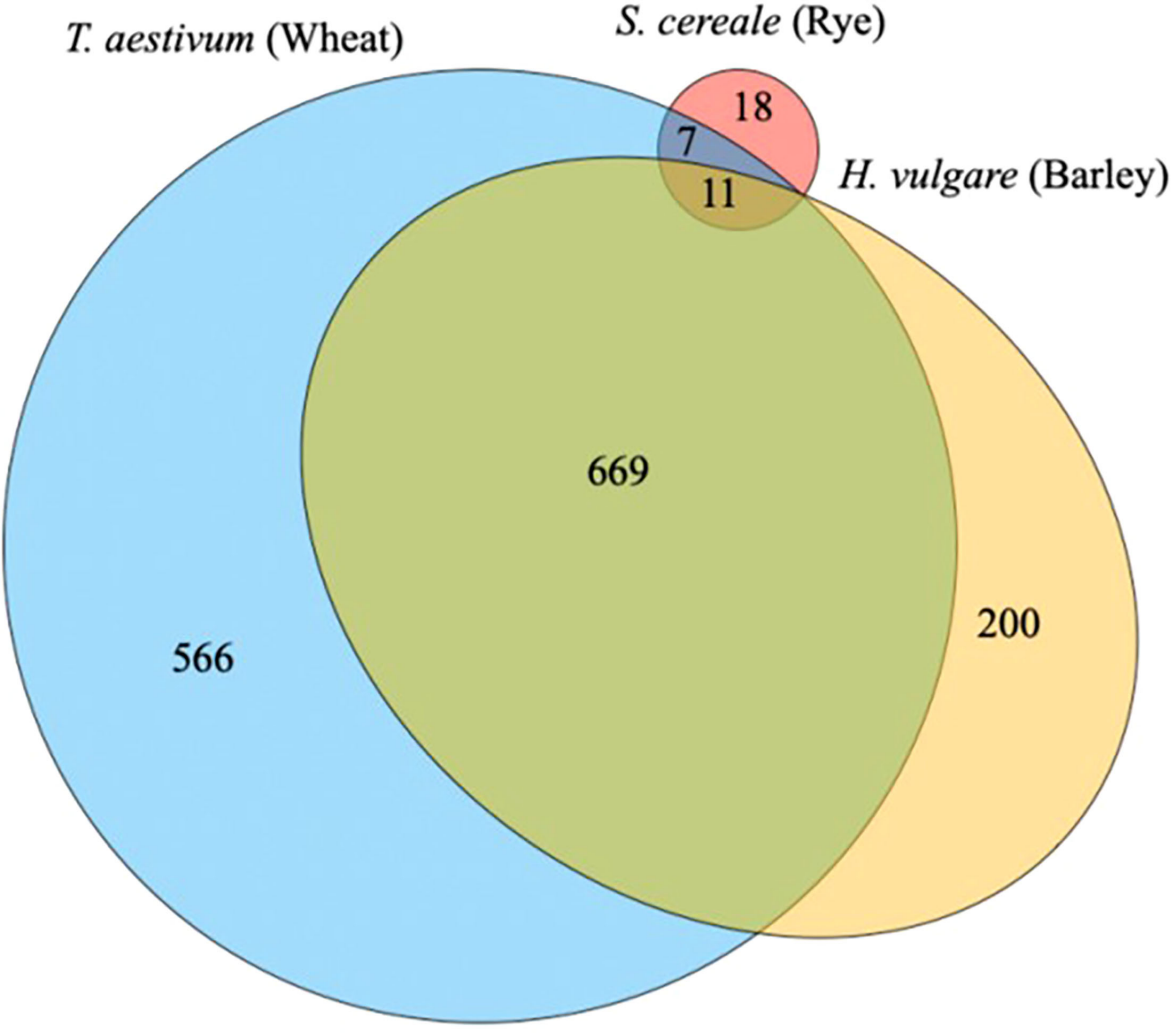
The distribution of proteins identified during malting between three species to group functional proteins in a wild relative of wheat, Thinopyrum intermedium, commercial named as Kernza (Marcus et al., 2022).

In the context of the malting and brewing industries, known databases to the main grain species such as barley, wheat, rice, corn, and sorghum would have the most peptides identified. The proteins from these species have been well defined based on decades of research for a range of different functionalities. If a lesser used grain type such as rye is tested, many of the peptides identified would mostly likely come from *Triticum aestivum* species (wheat) or a close related rye (*Secale cereale*) and compared with the barley (*Hordeum vulgare*) proteome. A recent study exploring a perennial wheat grass (*Thinopyrum intermedium*, Kernza), which does not have a specific database of known proteins, found peptides from wheat and barley as the most abundant (Marcus et al., 2022) (**Figure 6**). This is partially explained by the long evolution path from an original grass species. Many of the proteins found in our current grain species are functional to ensure survival and the next generation of seeds.

Typically in the proteomics results of grains, malt wort or beer, the most abundant proteins identified are major endosperm storage proteins (alcohol and alcohol with reducing agent soluble). As mentioned above, these proteins are a major portion of the total amount of protein. Other major proteins are usually endosperm-bound proteins such as beta-amylase, lipid transfer protein, and serine proteinase inhibitor (Serpin). These proteins are common to all grains as these are regulatory proteins required in grain development or during germination.

In wheat and barley, puroindoline (wheat) and hordoindolines (barley), respectively, are abundant as these are proposed to be involved in some bonding of the endosperm storage proteins to starch granules (Giroux and Morris, 1997; Fox et al., 2007). Other proteins (starch synthesis enzymes) identified can be those involved in starch synthesis during grain filling.

In the context of wort composition, proteomics can identify the 100 and possibly more than 1000 proteins in the wort, depending upon the malts and adjunct combinations. Proteomics allows exploration of every protein, from proteins expressed in grain, during germination, remaining through into final beer, making up the hundreds of grain-based proteins in beer. This profile of numerous proteins includes all the individual enzymes. However, there is no information on specific enzyme activity, just the presence of the hundreds of enzymes and their relative amount to all other proteins. However, it is possible to track the loss of some proteins and enzymes during the heating stages, i.e., kilning in malting, mashing/sparging, and boiling in brewing.

Another analysis of proteins, specifically the peptides produced by the action of proteomics, is peptidomics (Picariello et al., 2015). As the name suggests, specific peptides rather than in tack proteins are detected and is a complementary analysis to proteomics. This technology can identify peptides up to around 100 amino acids. To date, there has been only a single study using peptidomics in beer which explored the contribution of wheat in a single beer style, i.e., weissbiers. The research investigated the endosperm storage peptides responsible for gluten intolerance (Picariello et al., 2015). However, this technology will be complimentary with proteomics as the peptidomics can identify specific dipeptides and tripeptides malt, wort, and beer based on protein hydrolysis, during malting and mashing. Peptidomics could also be used to track assimilation of dipeptides during fermentation, compared with the residual dipeptides. In wheat and it relatives, as well as barley, a high amount of peptides with a proline could impact beer quality through proline-rich peptides bonding with polyphenols causing unwanted beer haze.

**Figure 7:**
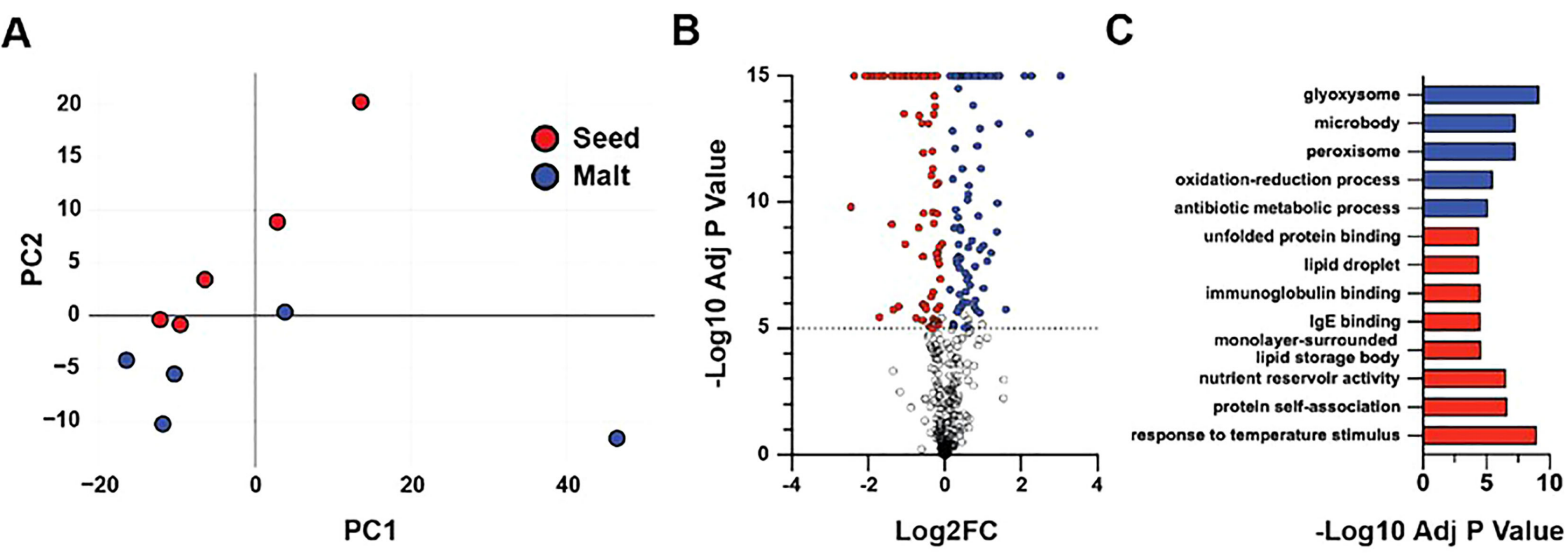
Changes in the sorghum proteome between grain and malt. **(A)** PCA of protein abundance normalized to the abundance of trypsin in each sample. **(B)** Volcano plot of the comparison of mature sorghum seeds and sorghum malt. Grey, not significantly different; red, significantly (p < 10−5) less abundant in malt; blue, significantly (p < 10−5) more abundant in malt. **(C)** Significantly enriched GO terms for proteins with significant differences in abundance comparing mature seeds and malt. Values are shown as –log2 of Bonferroni-corrected p values for GO terms, which were significantly enriched (p < 0.05) in proteins that were significantly more abundant in malt, blue; or significantly less abundant in malt, red (Kerr and Schulz BL, 2022).

**Figure 7** provides an example of changes in major protein functional groups from sorghum grain to sorghum malt.

## Conclusions

Through the advancement of different technologies, the grain, malting, and brewing industries will have more data than can be easily managed for day-to-day use. However, over time, understanding how to use a single ‘omics analysis or a mix of ‘omics analysis will inform breeders of specific genetic targets for crops to better handle climate change and give more consistent quality in changing production environments, or to improve the speed of modification during malting to reduce to costs of malting. Beer flavor and consistency of flavor will be improved with knowledge of metabolites, their source of origin (grain), or changes based on processing (malting and brewing). The challenges ahead are the integration of these technologies at industry levels, communication of the benefits to industry, and more research through industry and researcher collaborations.

## Author contributions

All authors contributed to the article and approved the submitted version.
